# Evaluation of the radiopacity of single-shade composite restorative materials using a digital radiography system

**DOI:** 10.1007/s00784-025-06453-8

**Published:** 2025-07-09

**Authors:** Işıl Doğruer, Merve Kütük Ömeroğlu, Burak Gümüştaş

**Affiliations:** 1 Istanbul Okan University, Faculty of Dentistry, Department of Restorative Dentistry, Istanbul, Turkey; 2 Istanbul University- Cerrahpasa, Faculty of Dentistry, Department of Restorative Dentistry, Istanbul, Turkey

**Keywords:** Composite resin, Single-shade, Radiopacity, Radiography

## Abstract

**Objectives:**

Recently, single-shade universal composite resins have been introduced to the market to simplify restorative procedures. The inorganic structures of dental composites may influence several properties, including radiopacity. Radiopacity plays a crucial role in clinical dentistry as it enables the detection of recurrent caries, marginal defects, and restoration overhangs through radiographic examination. The aim of this investigation was to assess the mean gray value and compare the radiopacity of different single-shade composite resins using digital image analysis.

**Materials and methods:**

Seven single-shade universal composite resins (Omnichroma, Charisma Topaz One, Vitra Aps Unique, Admira Fusion x-tra universal, X-tra Fill, Essentia Universal and ZenChroma) and one multi-shade universal composite resin (Optishade) were used. The enamel-dentin segment, a typical aluminum (Al) step-wedge (1–10 mm), and the samples were placed on a phosphor imaging plate. For each image, the MGV and the standard deviation of the grey values for three different regions were calculated and the mean of these three values was recorded.

**Results:**

mmAI measurements of the study groups were found to be statistically significant different (*p* < 0.05). The differences were found between Charisma one and Voco xtrafil groups and between Enamel, Dentin and Zenchroma groups. Also, differences were observed between Dentin and Vittra aps unq groups. The radiopacity values of all materials tested were found to meet the requirements of ISO.

**Conclusions:**

It is found that Glass particles containing composites have similar radiopacity as enamel due to their low atomic weight. Zirconium particles containing composites are more radiopaque than glass particles containing composites because of their higher atomic weight.

## Introduction

Dental composites are used in layers of different colors and different translucency to mimic natural teeth for a better aesthetic fit [1]. Recently, single-shade universal composite resins have been introduced to simplify restorative procedures. These materials are engineered to match all 16 VITA classical shades (A1–D4) by leveraging advanced filler technologies and resin matrix modifications, thereby minimizing aesthetic discrepancies between the restoration and the natural tooth structure.” [2–4].

Inorganic structures of dental composites may affect the dental composites properties in many ways such as radiopacity, mechanical strength, depth of the cure, translucency and other factors. Since barium, ytterbium, aluminum, or strontium containing fillers may contribute to radiopacity, filler content could affect a composite resin’s radiopacity [5]. Radiopacifying agents commonly incorporated in dental restorative materials include zinc oxide (ZnO), zirconium oxide (ZrO_2_), barium sulfate (BaSO_4_), barium oxide (BaO), yttrium fluoride (YF_3_), strontium oxide (SrO), bismuth oxide (Bi_2_O_3_), lanthanum oxide (La_2_O_3_), and ytterbium trifluoride (YbF_3_). The American Dental Association describes radiopacity as one of the five essential requirements for dental materials [6, 7]. There are several reasons why radiopacity is a clinically important characteristic. It is effective in detecting secondary caries underlying restorations, which is a significant cause of restorative failure. It allows for the identification of gaps and exposed margins in the cervical area of proximal restorations, as well as voids and cavities within the restoration [8–10]. At following recall appointments, it can assess the restoration’s integrity. Additionally, it can assess proximal shapes, connections with neighboring teeth or restorations, and excessive proximity to the pulp chamber [11–14].

There are various commercially available dental restorative materials that have different degrees of radiopacity. Existing research on radiopacity typically compare a material with enamel, dentin, or aluminum. Numerous researches within the literature have evaluated the radiopacity of dental materials by comparing them to an equivalent thickness of pure aluminum (Al) in millimeters. This is achieved by constructing a reference calibration curve based on the radiopacity data of aluminum. As specified by the American Dental Association’s (ADA) Specifications [15], radiopacities of materials that are one millimeter thick must at least match those of one-millimeter-thick pure aluminum, which is also the same as one-millimeter-thick dentin tissue [16].

Due to their unique structural characteristics, monochromatic composites differ from conventional composites, and as a result, their radiopacity has been evaluated in only a limited number of studies. These materials are predominantly used in anterior esthetic restorations, where patients are generally more likely to attend routine follow-up appointments. In this context, the radiopacity of single-shade composites gains clinical relevance, as radiographs taken during control visits play a crucial role in the detection of secondary caries and the overall assessment of restoration integrity.

The majority of the literature on dental materials’ radiopacities discusses flowable composite resins and materials intended for posterior application [17–23]. Due to their unique structural characteristics, monochromatic composites differ from conventional composites, and as a result, their radiopacity has been evaluated in only a limited number of studies [24, 25]. These materials are predominantly used in anterior esthetic restorations, where patients are generally more likely to attend routine follow-up appointments. In this context, the radiopacity of single-shade composites gains clinical relevance, as radiographs taken during control visits play a crucial role in the detection of secondary caries and the overall assessment of restoration integrity. Differences in radiopacity levels may occur due to the compositional properties of different monochromatic composite resins. Restorative material choices of clinicians may be affected by these differences [8, 21, 26].

Given their widespread use in both anterior and posterior regions due to their shade-matching capabilities, single-shade composites must exhibit adequate radiopacity to ensure accurate detection of restoration margins, recurrent caries, or voids in radiographic evaluations. This study aimed to assess the mean gray value and compare the radiopacity of single-shade composite resins through digital image analysis. The null hypothesis was that there would be no significant difference in radiopacity either between the tested materials and tooth tissues or among the materials used in the study.

## Materials and methods

### Sample preparation

In this study, seven different single-shade composite resins and one multi-shade composite resin were used. Table 1 provides an overview of the composite resins included in the research and their respective properties. A comprehensive power analysis was conducted using G*Power 3.1 software (Heinrich Heine University, Dusseldorf, Germany), drawing upon insights from a comparable study [25]. This analysis established a minimum required sample size of 5 specimens for each experimental group in this investigation. The statistical parameters employed were an alpha probability of error set at 0.05 and a statistical power of 0.80 (effect size 0.65). The disc dimensions of 5 mm in diameter and 1 mm in thickness were selected to standardize the specimen size based on the tip diameter of the light-curing unit and to ensure compatibility with the 1 mm thickness of the aluminum step wedge used as a radiopacity reference. Five disc-shaped samples (5 mm x 1 mm) were prepared for each tested composite material (*n* = 5) using a teflon mold. Glass slides were positioned beneath the molds to guarantee that the materials had a sufficiently flat surface. The resins were filled in the molds in the form of a single layer to prepare the resin composite specimens. Transparent strips were used to smooth their upper surfaces before polymerization. Subsequently, the active end of the light source (3 M Elipar DeepCure-S, 1470 mW/cm², 3 M ESPE, USA) was placed at the center of the specimen and light-cured for 20 s. All composite specimens were stored in distilled water at 37 °C for 24 h prior to the radiopacity evaluation.

**Table 1:** The compositions and properties of the tested materials

Brand name	Matrix	Filler composition/size	Filler w/v%	Manufacturer	Lot No.
Omnichroma	UDMA, TEGDMA	Uniform sized supra-nano spherical filler (260 nm spherical SiO2–ZrO2)	79/68	Tokuyama Dental Corporation, Tokyo, Japan	2652
Charisma Topaz one	UDMA, TCD-DI-HEA, TEGDMA	Barium aluminum fluoride glass filler of 0.02–2 μm, 5 vol% pyrogenic silicon dioxide filler of 0.02–0.07 μm	81/64	Kulzer, Hanau, Germany	N010208
Vittra APS unique	UDMA, TEGDMA	Zirconia charge, silica (200 nm)	82/72	FGM Dental, Joinville, SC, Brasil	230921
Admira Fusion x-tra universal	ORMOCER	photoinitiators, pigments, barium aluminum borosilicate glass, pyrogenic sílica (20–50 nm)	84 wt%	VOCO, Germany	2340432
X-tra Fill	Bis-GMA, UDMA, TEGDMA	Inorganic filler (Bariumaluminium silicate, fumed silica, pigments)	86 wt%	VOCO, Germany	2343825
Essentia	UDMA, BIS- MEPP, BIS‐EMA, BIS‐ GMA, TEGDMA	PPF (17 μm): strontium glass (400 nm), lanthanide fluoride (100 nm), fumed silica (16 nm) FAISi glass (850 nm)	81/ 65	GC Europe, Leuven, Belgium	2301181
ZenChroma	UDMA, Bis-GMA, TEMDMA	Glass powder, silicon dioxide, inorganic filler (0.005–3.0 μm)	75/53	President Dental GmbH, Allershausen, Germany	2023002012
Optishade	Bis-EMA, Bis-GMA, TEGDMA	PPF, BaO–Al2O3–SiO2, silica, F3Yb	81/64.5	Kerr Dental, California, USA	8729311

The Ethics Committee of Istanbul Okan University approved the utilization of a molar tooth that had been extracted and was free of decay, cracks, or deformities (decision no. 172, dated 10.01.2024). A 1 mm thick containing enamel-dentin samples were obtained from the third molar using a low-speed diamond saw for precise sectioning (Isomet Low Speed Saw 1000; Buehler, Lake Buff, IL, USA). A digital caliper was used to verify that the thickness of the material and tooth sample was within 1.00 ± 0.01 mm.

### Radiopacity measurement

**Fig. 1:**
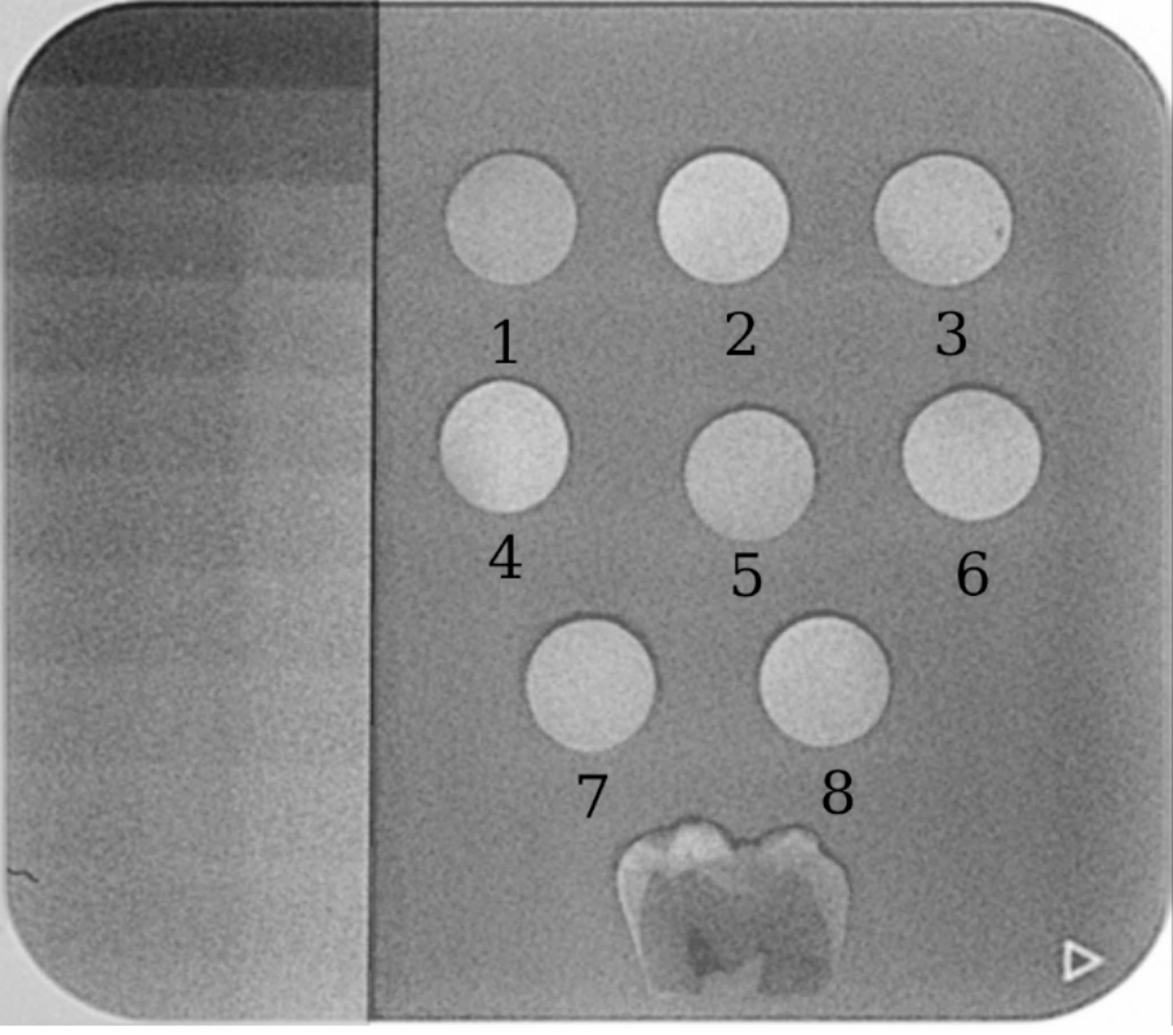
Radiograph of composite specimens, tooth slice and aluminium stepwedge. 1. Omnichroma 2. Charisma Topaz one 3. Admira Fusion x-tra 4. X-tra Fill 5. ZenChroma 6. Optishade 7. Vittra APS unique 8. Essentia

A size 4 C (48 mm x 54 mm) phosphor imaging plate (Planmeca Dental, Helsinki, Finland) was used to position the specimens, a standard aluminum (Al) step-wedge (1–10 mm), and the enamel-dentin section for imaging (Fig. 1). With a focal spot distance of 30 cm, the dental X-ray device (Planmeca Dental, Helsinki, Finland) was set to 60 kV and 7 mA, with an exposure time of 0.13 s. to capture images of the specimens. A scanner (Planmeca Dental, Helsinki, Finland) was employed to digitize the phosphor plate, and five radiographs were stored in 8-bit TIFF format after being transferred to the computer. The mean gray values (MGVs) of both the specimens and the step-wedge were subsequently analyzed using Photoshop CS6 software (Adobe Systems, San Jose, CA, USA). This approach is widely recognized due to its accessibility and cost-efficiency, requiring no additional specialized equipment, and it is frequently utilized for assessing the radiopacity of restorative materials. For each radiograph, three separate measurements were taken from both the specimens and each aluminum step within a 1 mm² area (a 10 × 10 pixel) region of interest (ROI)), and the mean of these measurements was calculated and documented.

The mean gray value (MGV) and the standard deviation of the gray values were calculated for each region of interest (ROI), and the average of the three regions was determined for each specimen in every radiograph (Fig. 1). The MGVs of the stepwedge in each image were utilized to generate a calibration curve for each radiograph. The CurveExpert version 1.4 program (Hyams D.G., Starkville, MS, USA) was employed to convert the mean gray value of the material into millimeters of aluminum equivalent. All evaluations were conducted by a single operator who was blinded to the identity of the materials.

### Statistical analysis

SPSS version 25.0 (IBM SPSS Statistics, Chicago, IL, USA) was used in all statistical analyses. Shapiro Wilk test was used to check the normality assumption. Differences between three or more independent groups where the normality assumption was not valid was examined with Kruskal Wallis test. The group or groups that created the difference was found with Post Hoc Bonferroni test.

## Results

**Fig. 2:**
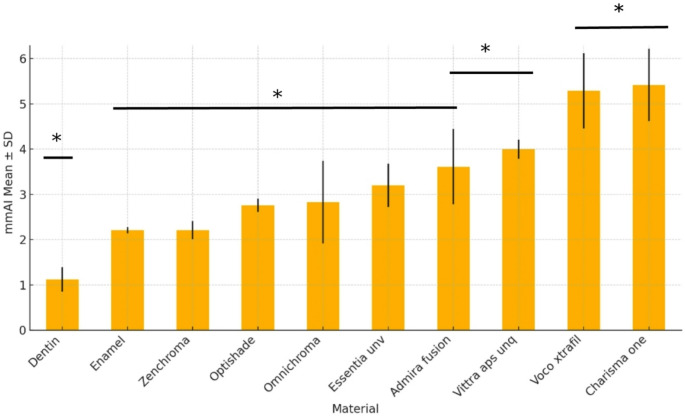
Distribution of mmAI values across study groups (sorted ascending). Error bars represent standard deviations. Statistically significant differences were observed among groups (Kruskal–Wallis test *p* < 0.001, *p < 0.05)

mmAI measurements of the study groups were compared with Kruskal-Wallis test and a statistically significant difference was found (*p* < 0.05). The highest levels of radiopacity among the composite materials were exhibited by Charisma One (5,42 ± 0,80 mmAl) and Voco xtrafil (5,29 ± 0,83 mmAl), with no significant difference between the two materials (*p* < 0,05). Meanwhile, the lowest level of radiopacity among the tested materials was presented by Zenchroma (2,21 ± 0,2 mmAl) (Table 2; Fig. 2). All the tested composite resins exhibited higher radiopacity values than enamel (2,21 ± 0,07 mmAl) and dentin (1,12 ± 0,27mmAl) (*p* < 0.05), except for Zenchroma, which presented a equal radiopacity value with enamel (*p* > 0.05).

**Table 2:** Distribution and comparison of MmAI measurements according to study groups

Materials	Min.-Max.	Mean ± sd (median)	Test Statistics	*p*
Enamel	2,13 − 2,29	2,21 ± 0,07(2,24) ^c^	46,425	< 0,001*
Dentin	0,84 − 1,47	1,12 ± 0,27(1,17) ^d^		
Omnichroma	1,38 − 3,81	2,83 ± 0,91(2,93) ^c^		
Charisma one	4,63 − 6,53	5,42 ± 0,80(4,99) ^a^		
Admira fusion	2,28 − 4,23	3,61 ± 0,83(4,09) ^b, c^		
Voco xtrafil	4,16 − 6,23	5,29 ± 0,83(5,47) ^a^		
Zenchroma	1,99 − 2,50	2,21 ± 0,2(2,23) ^c^		
Optishade	2,64 − 2,98	2,76 ± 0,15(2,69) ^c^		
Vittra aps unq	3,64 − 4,13	4,00 ± 0,21(4,09) ^b^		
Essentia unv	2,48 − 3,65	3,20 ± 0,48(3,38) ^c^		

*p < 0,05

**Fig. 3:**
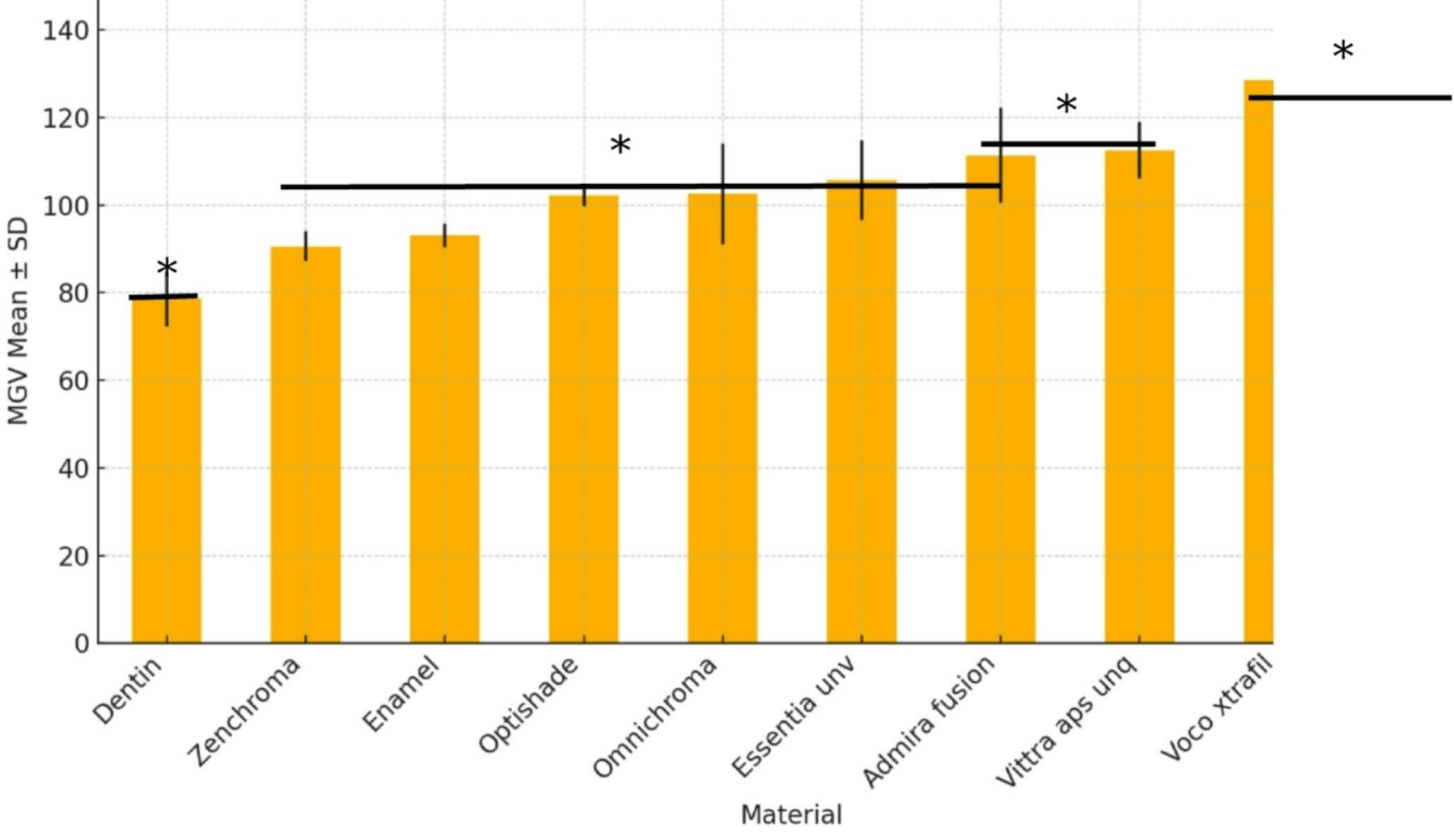
Distribution of MGV values across study groups (sorted ascending). Error bars represent standard deviations. Statistically significant differences were found among groups (Kruskal–Wallis test *p* < 0.001, *p < 0.05)

The Kruskal-Wallis test was used to compare the MGV measurements across the study groups, revealing a statistically significant difference (*p* < 0.05). The highest levels of radiopacity among the composite materials were exhibited by Charisma One (130,35 ± 10,04 MGV) and Voco xtrafil (128,54 ± 4,43 MGV), with no significant difference between the two materials (*p* < 0,05). Meanwhile, the lowest level of radiopacity among the tested materials was presented by Zenchroma (90,61 ± 3,35 MGV) (Table 3; Fig. 3).

**Table 3:** Distribution and comparison of MGV measurements according to study groups

Materials	Min.-Max.	Mean ± sd (median)	Test Statistics	*p*
Enamel	90,04–96,7	93,14 ± 2,71(92,94) ^c^	44,343	< 0,001*
Dentin	70,01–87,48	78,64 ± 6,36(78,93) ^d^		
Omnıchroma	82,2-109,04	102,53 ± 11,45(106,63) ^c^		
Charısma one	116,71–140,75	130,35 ± 10,04(131,26) ^a^		
Admıra fusion	92,67–119,65	111,34 ± 10,84(113,44) ^b, c^		
Voco xtrafil	122,3-134,25	128,54 ± 4,43(128,65) ^a^		
Zenchroma	87,62–95,32	90,61 ± 3,35(89,82) ^c^		
Optishade	100,17–105,38	102,15 ± 2,36(101,17) ^c^		
Vittra aps unq	102,14–117,62	112,5 ± 6,37(114,98) ^b^		
Essentia unv	94,32–114,57	105,66 ± 9,02(110,22) ^c^		

*p < 0,05

## Discussion

In our study, all of the composite resins evaluated had higher mm Al and MGV values than dentin and therefore provided the ISO 4049 standard. Radiopacity levels close to or higher than enamel, except for the ZC group (2.21 ± 0.2 mm Al) were found in the tested composite resins. Therefore, our null hypothesis “there would be no significant difference in radiopacity either between the tested materials and tooth tissues or among the materials used in the study.” was partially rejected.

According to sources, a radiopacity value greater than that of enamel is the best acceptable value for the detection of secondary caries on radiography. These data provide a great advantage for the tested materials in the detection of faulty/incomplete restoration or secondary caries under the restoration and prevent the misinterpretation of restorations made with these materials as dental caries in dental radiographic evaluation [9, 27].

A prior study indicated that composite resins with approximately 70% filler volume can achieve radiopacity levels greater than enamel when the radiopaque element content in the fillers exceeds around 20%. In the current investigation, seven of the eight composite resins analyzed demonstrated radiopacity values higher than enamel. However, all except for ZC (with a filler load of 75%) had filler contents exceeding 75%, and ZC displayed the lowest radiopacity value [28].

Many studies have obtained supporting results that the inorganic composition of a composite material is one of the factors affecting the radiopacity of the composite material [18, 29–32]. To impart radiopaque properties, manufacturers add inorganic particles of elements with high atomic numbers such as Bi (*Z* = 83), Yb (*Z* = 70), La (*Z* = 57), Ba (*Z* = 56), Zr (Z = 40), Y (*Z* = 39), Sr (*Z* = 38), and Zn (*Z* = 30) to the composition of composite resins to increase the radiopacity properties of the materials. In our study, in parallel with these data, the radiopacity value of barium-containing CTO and XF group was found to be higher compared to other composites. Charisma Topaz One showed the highest radiopacity in this study, followed by X-tra Fil. Although the weight% of X-tra Fil (86w%) was higher than Charisma Topaz One (81w%), the radiopacity of X-tra Fil was lower. Volume percent of X-tra Fil (70v%) was higher than Charisma Topaz One (64v%), the radiopacity of X-tra Fil was lower. Both composite materials contain barium and aluminum, which have high atomic number, but addition of silicon dioxide to composition of organic matrix instead of fumed silica increase the radiopacity of CTO, and the results of our study are in line with the results of Ağaccıoğlu and Yılmaz’s study [24]. Therefore, the null hypothesis stating that “there would be no significant difference in radiopacity either between the tested materials and tooth tissues or among the materials used in the study” was completely rejected.

All composite resins tested in this study exhibited higher mean gray values (MGVs) and millimeter aluminum (mm Al) values than dentin (1.12 ± 0.27), as shown in Table 2, thus meeting the ISO 4049 standard. These results indicate a beneficial characteristic for the materials, reducing potential misinterpretations in dental radiography when evaluating restorations as possible caries. However, it has been noted that restorative materials should possess greater radiopacity than enamel to allow for clear differentiation between restorations and subsequent caries. Except for the ZC group (2.21 ± 0.2 mm Al), which showed radiopacity values closer to enamel, the other composite resins displayed higher radiopacity levels than enamel (2.21 ± 0.07 mm Al) in this study. This result can be interpreted as the low atomic weights of the glass particles in its content and the low volume of inorganic particles compared to other composites, which is why its radiopacity shows similar properties to enamel. On the other hand, silica is a low atomic number element (SiO_2_ = 40) present in the filler content of resin composites, which may account for the relatively low radiopacity observed in the silica-containing resin groups (ZC, OS, OC) in the present study. VAU with zirconia filler showed less radiopacity than CTO with barium aluminum filler, despite having more filler by weight and volume. As a result of their study, Xu et al. suggested that resin composites can show high radiopacity with higher atomic number elements even at lower filler percentage in volume and weight [29]. In parallel with this result, the result of our study supports the conclusion that the atomic weight of inorganic filler particles in the material content affects the radiopacity before the amount of inorganic filler particles.

Assessing the radiopacity of restorative materials is crucial for ensuring accurate interpretation of radiographs and effective clinical decision-making. However, it is equally important to consider the radiopacity of materials used beneath restorations, such as liners and adhesive agents. Multiple studies have demonstrated that many dental adhesives and liners possess radiopacity levels lower than dentin, which can complicate radiographic diagnostics by mimicking carious lesions or masking marginal defects [30–32]. For instance, Dawoud et al. (2025) emphasized the clinical value of using radiopaque adhesives in cervical restorations to avoid misinterpretation of adhesive layers as caries [30]. Similarly, Oztas et al. (2012) and de Moraes Porto et al. (2014) found considerable variation in the radiopacity of current bonding systems, some falling below the radiodensity threshold of dentin. Furthermore, studies evaluating liners and base materials [31, 32], such as those by Lachowski et al. (2013) and Yaylacı et al. (2021) [8, 33], reported that several commonly used materials displayed insufficient radiopacity, raising concerns over their diagnostic reliability in radiographic assessments. These findings collectively highlight the necessity of incorporating radiopacity as a key selection criterion not only for restorative materials but also for liners and adhesives in routine clinical practice. Therefore, a comprehensive assessment of the entire restoration is required. Recent evaluations support this concern by indicating that adhesives with low radiopacity may lead to diagnostic errors on clinical radiographs, particularly in deep class II restorations. For example, Bazerbashi et al. (2023), in their study investigating the prevalence and characteristics of radiolucent zones associated with class II composite restorations, emphasized that adhesives with low radiopacity could contribute to diagnostic ambiguity around restoration margins [34].

It should also be noted that the radiopacity values presented in this study were obtained by evaluating the composite resins alone. However, in clinical conditions, other materials such as adhesives and liners may be present beneath or adjacent to the restorative material. Since these additional materials were not included in the radiographic analysis, the results may not entirely represent the radiopacity of restorations as they appear in real clinical settings. Future studies incorporating the full layering technique, including adhesives and base materials, are recommended to better simulate intraoral conditions.

## Conclusion

Within the limitations of the study we can conclude that all tested materials had radiopacity values that provide ISO standards. Composites containing barium with aluminum inorganic fillers such as Charisma One and Voco xtrafil tends to be more radiopaque than composites containing barium oxide inorganic filler alone. ZenChroma which contains only glass particles organic filler have similar radiopacity as enamel tissue. Vittra APS unique composite contains zirconium inorganic fillers which is less radiopaque than composites containing barium with aluminum inorganic fillers. Optishade and Essentia composites containing prepolymerized filler with aluminum show similar properties although Essentia composite doesn’t contain barium inorganic filler.

## Data Availability

No datasets were generated or analysed during the current study.
